# Ruddlesden–Popper 2D perovskites of type (C_6_H_9_C_2_H_4_NH_3_)_2_(CH_3_NH_3_)_n−1_Pb_n_I_3n+1_ (n = 1–4) for optoelectronic applications

**DOI:** 10.1038/s41598-022-06108-8

**Published:** 2022-02-09

**Authors:** Mohammad Rahil, Rashid Malik Ansari, Chandra Prakash, S. S. Islam, Ambesh Dixit, Shahab Ahmad

**Affiliations:** 1 Advanced Energy Materials Group, Department of Physics, Indian Institute of Technology Jodhpur, Jodhpur, Rajasthan 342037 India; 2 Centre for Nanoscience and Nanotechnology, Jamia Millia Islamia (A Central University), New Delhi, 110025 India; 3 Department of Physics, Indian Institute of Technology Jodhpur, Jodhpur, Rajasthan 342037 India

**Keywords:** Materials for devices, Two-dimensional materials, Lasers, LEDs and light sources

## Abstract

Ruddlesden–Popper (RP) phase metal halide organo perovskites are being extensively studied due to their quasi-two dimensional (2D) nature which makes them an excellent material for several optoelectronic device applications such as solar cells, photo-detectors, light emitting diodes (LEDs), lasers etc. While most of reports show use of linear carbon chain based organic moiety, such as n-Butylamine, as organic spacer in RP perovskite crystal structure, here we report a new series of quasi 2D perovskites with a ring type cyclic carbon group as organic spacer forming RP perovskite of type (CH)_2_(MA)_n−1_Pb_n_I_3n+1_; CH = 2-(1-Cyclohexenyl)ethylamine; MA = Methylamine). This work highlights the synthesis, structural, thermal, optical and optoelectronic characterizations for the new RP perovskite series n = 1–4. The demonstrated RP perovskite of type for n = 1–4 have shown formation of highly crystalline thin films with alternate stacking of organic and inorganic layers, where the order of PbI_6_ octahedron layering are controlled by n-value, and shown uniform direct bandgap tunable from 2.51 eV (n = 1) to 1.92 eV (n = 4). The PL lifetime measurements supported the fact that lifetime of charge carriers increase with n-value of RP perovskites [154 ps (n = 1) to 336 ps (n = 4)]. Thermogravimetric analysis (TGA) showed highly stable nature of reported RP perovskites with linear increase in phase transition temperatures from 257 °C (n = 1) to 270 °C (n = 4). Scanning electron microscopy (SEM) and energy dispersive X-ray analysis (EDAX) are used to investigate the surface morphology and elemental compositions of thin films. In addition, the photodetectors fabricated for the series using (CH)_2_(MA)_n−1_Pb_n_I_3n+1_ RP perovskite as active absorbing layer and without any charge transport layers, shown sharp photocurrent response from 17 nA/cm^2^ for n = 1 to 70 nA/cm^2^ for n = 4, under zero bias and low power illumination conditions (470 nm LED, 1.5 mW/cm^2^). Furthermore, for lowest bandgap RP perovskite n = 4, (CH)_2_MA_3_Pb_4_I_13_ the photodetector showed maximum photocurrent density of ~ 508 nA/cm^2^ at 3 V under similar illumination condition, thus giving fairly large responsivity (46.65 mA/W). Our investigations show that 2-(1-Cyclohexenyl)ethylamine based RP perovskites can be potential solution processed semiconducting materials for optoelectronic applications such as photo-detectors, solar cells, LEDs, photobatteries etc.

## Introduction

In the past decade various three dimensional (3D) metal halide perovskite semiconductors particularly methylamine lead iodide CH_3_NH_3_PbI_3_ have gained lots of attention due to their excellent electronic and optical properties like large charge carrier lifetimes (~ 570 ns), long diffusion lengths (~ 10 µm), high mobilities (~ 67 cm^2^/V-sec ), low non-radiative recombination rates, large light absorption coefficients (~ 10^5^ cm^−1^) and low direct bandgaps in the visible range of spectrum (~ 1.6 eV)^1–7^. These 3D metal halide perovskites have been widely demonstrated for various optoelectronic applications such as perovskite solar cells (PSCs), light-emitting diodes (LEDs), photodetectors (PDs), nanolasers, field-effect transistors (TFTs), solar water splitting, photo-batteries etc.^8–14^. However in spite of excellent optoelectronic properties, these 3D metal halide perovskites suffer from the inherent structural instability in the ambient atmospheric conditions, which limit their utilizations in commercial devices^15,16^. Contrary to the bulk perovskites, the low-dimensional counterpart such as 2D perovskites of type butylamine lead iodide (C_4_H_9_NH_3_)_2_PbI_4_ are also studied for their unique strong room-temperature exciton properties, structural flexibility and improved stabilities^17,18^. 2D metal halide perovskites forms naturally self-assembled quantum well type structures which leads to the formation of excitons with large binding energies (~ 300 meV), owing to the dielectric and quantum confinement effects^19,20^. The large binding energies results in reduced charge carrier lifetimes (~ 502 ps) and diffusion lengths (~ 1.8 µm), which limits their usage in wide range of optoelectronic devices^21^.

More recently, the Ruddlesden–Popper (RP) phase metal halide perovskites are gaining lot of attention due to their well-controlled bandgaps, unique structural flexibility and improved stability compared to the 3D metal halide perovskites^14,22^. These RP phase perovskite materials are derivative of 3D perovskites and denoted by the general formula of (RNH_3_)_2_(MA)_n−1_M_n_X_3n+1_ where RNH_3_ is a large organic cation, MA is methylamine which acts as small organic cation, the integer *n* represent number of metal halide octahedron MX_6_ layers between adjacent insulating organic layers comprising of RNH_3_ and MA, M^2+^ is a divalent metal cation and X^−^ is a halide anion^17,23–26^. Thus, structurally RP perovskites forms a quasi-dimensional (2D + 3D) perovskite structure due to presence of both pure 2D and 3D phases. Hence the properties of these solution-processed metal halide RP perovskite semiconductors show strong dependence on *n* value which determines the extent of 2D and 3D phase present in the perovskite structure. For n = 1, a pure 2D perovskite structure of type (RNH_3_)_2_MX_4_ can be achieved and for n = ∞, a close to 3D perovskite structure can be achieved. Thus RP perovskites demonstrate optoelectronic properties which comprise of wide range of properties from 2 to 3D perovskites^1,18,27–29^. In 2014, Smith et al. demonstrated the use of quasi-2D RP perovskites (PEA)_2_(MA)_2_Pb_3_I_10_ (where PEA is phenylethylamine) as a light absorber in PSCs, and reported photo-conversion efficiency (PCE) of 4.76% ^30^. Further, In 2015 Cao et al. successfully replaced organic spacer PEA by butylamine (BA, C_4_H_9_NH_2_) to get (BA)_2_(MA)_2_Pb_3_I_10_ perovskite in PSC, however the performance of solar cells remained approximately similar (PCE ~ 4.02%)^31^. Meanwhile in 2019, Liu et al. reported RP perovskite light absorber layer based hetero-structure PSC with an impressive PCE of ~ 20.6% and superior stability of more than 1000 h in humid air under simulated sunlight conditions^32,33^. The utmost reason behind the enhanced stability of quasi 2D metal halide perovskites is attributed to the hydrophobic organic side chains which resists interaction of moisture with MA (soluble in H_2_O) which further helps in maintaining the layered structure^30,34^, as well as the van der Waals interactions between the capping organic molecules^35^.

Apart from the application of RP metal halide perovskites in PSCs, these hybrid materials are also been studied for other optoelectronic devices such as light emitting diodes and photodetectors. In 2016, Yuan et al. observed funneling effect in (PEA)_2_(MA)_n-1_Pb_n_I_3n+1_ type RP perovskite and demonstrated their use in LEDs, as an emission layer, which has shown high external quantum efficiency (EQE) of 8.8%^36^. In a very short span of time the EQE of quasi-2D perovskites based LEDs has reached around 20.1% for near-infrared^37^, 15.5% for green light^38^, and 6.2% for blue light emissions^39^. Apart from electroluminescent devices, in 2016 Zhou et al., reported first RP metal halide perovskite based photodetector, fabricated in lateral two electrode configuration, with (BA)_2_(MA)_n-1_Pb_n_I_3n+1_ (n = 1–3) as the absorbing layer^40^. The best performing photodetector has demonstrated high responsivity (R_ph_) of 12.78 mA/W and large On–Off ratio (I_photo_/I_dark_) of 1.0 × 10^3^ with an incident light intensity of 3.0 mW/cm^2^ and bias of 30 V. Further in 2018, Dong et al. reported novel photodetectors series where they observed improved responsitivity by replacement of linear *n*-BA (n-butylamine, n-C_4_H_9_NH_2_) organic cation with iBA (iso-butylamine, C_4_H_9_NH_2_) forming (iBA)_2_(MA)_n-1_Pb_n_I_3n+1_ as the absorbing layer. These photodetectors have shown responsitivity of 4.78, 25.81, 75.20, and 71.11 mA/W for n = 1, n = 2, n = 3 and n = 4 respectively, while n = 4 (iBA)_2_(MA)_3_Pb_4_I_13_ perovskite film grown through hot casting method, shown highest responsivity ~ 117.09 mA/W, large On − Off ratio ~ 4.0 × 10^2^ under low bias of 1.5 V in a lateral two electrode configuration^41^. In 2019 Liu et al., represented the effect of tailoring the large organic spacer cation on the optoelectronic properties of RP lead iodide n = 1 perovskites^42^ by fabricating (BA)_2_PbI_4_ (BA: butylamine), (HA)_2_PbI_4_ (HA: hexylamine) and (OA)_2_PbI_4_ (OA: octylamine) and investigated their optical, structural, and optoelectronic properties. A decrease in the dark current and increasee in the photocurrent is observed with increase in the length of organic chains. In 2018 Quarti et.al., revealed that the size and type of organic moiety affect the energy band gap and the effective masses of charges due to the distortion of PbI_6_ octahedra sheet^25,43^.

Though in the recent past most of the RP metal halide perovskites are studied for the linear carbon chain based organic spacers, such as butylamine based RP perovskites (BA)_2_(MA)_n−1_Pb_n_I_3n+1_ (n = 1–4) were extensively studies by Kanatzidis and co-workers^23^, and few reports are present on the use of cyclic carbon group based organic spacers. It has been recently demonstrated that cyclic carbon groups such as phenylethylamine (PEA = C_6_H_5_C_2_H_4_NH_2_) or cyclohexylethylamine (CH = C_6_H_9_C_2_H_4_NH_2_) based pure 2D perovskites show much improved optical and optoelectronic properties^10,18,19,36,44,45^. This is attributed to the fact that the large sized organic cation not only work as an insulating deterrent that restricted the charge carrier in two dimensional plane, but also as dielectric regulators that ascertain the electrostatic forces applied to the carriers^46,47^. Recently, Ghosh et. al., performed non-adiabatic molecular dynamics and time domain DFT to observe the carrier recombination processes in lead bromide based 2D perovskites with linear (n-butylammonium, (BA)_2_PbBr_4_) and cyclic (phenylethylammonium, (PEA)_2_PbBr_4_) organic cations^48^, where the reduced structural fluctuations are found to be responsible for large carrier lifetime and narrower emission line width in (PEA)_2_PbBr_4_ compare to (BA)_2_PbBr_4_.

**Figure 1:**
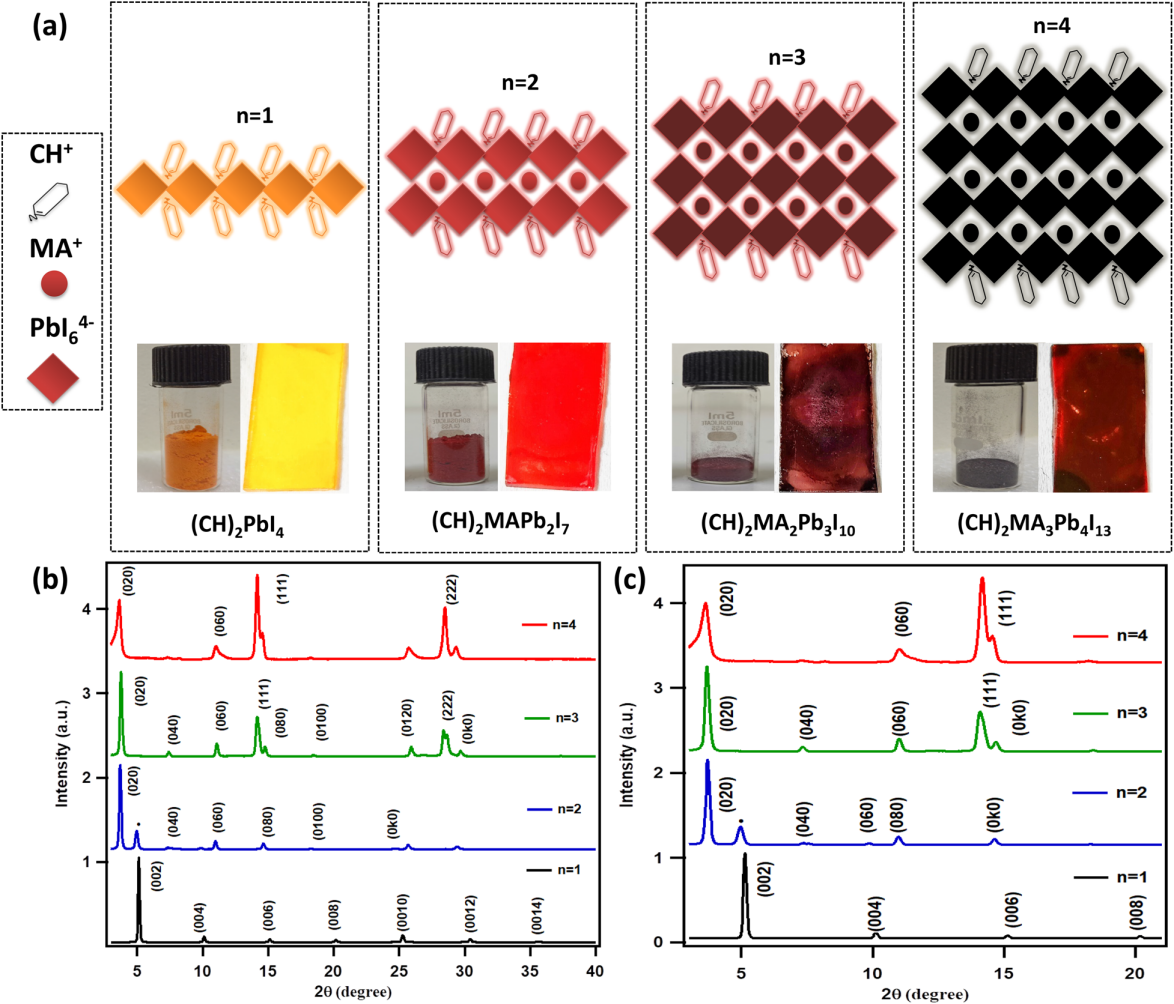
(**a**) Schematic illustration of the crystal structure of layered perovskite materials with digital camera image of powder and thin films of (CH)_2_(MA)_n−1_Pb_n_I_3n+1_ (n = 1, 2, 3 and 4), (**b**) X-ray diffraction (XRD) patterns of (CH)_2_(MA)_n−1_Pb_n_I_3n+1_ (n = 1, 2, 3 and 4) perovskite thin films, (**c**) Zoomed XRD plots of (CH)_2_(MA)_n−1_Pb_n_I_3n+1_ (n = 1, 2, 3 and 4) perovskites. All perovskite thin films are fabricated on glass substrates.

In this paper, we are reporting the synthesis and optoelectronic studies of new quasi-2D perovskites based on 2-(1-Cyclohexenyl)ethylamine (C_6_H_9_C_2_H_4_NH_2_^+^, hereafter CH) as organic spacer in the RP phase lead iodide perovskite of type (CH)_2_(MA)_n-1_Pb_n_I_3n+1_ with n = 1–4, namely (CH)_2_PbI_4_ (n = 1)_,_ (CH)_2_(MA)Pb_2_I_7_ (n = 2)_,_ (CH)_2_(MA)_2_Pb_3_I_10_ (n = 3)_,_ and (CH)_2_(MA)_3_Pb_4_I_13_ (n = 4). As mentioned above, the purpose for choosing CH organic spacer in RP metal halide perovskites is largely because the CH organic moiety is known to improve the dielectric confinement in the layered perovskites as well as the moisture resistance, hence improves overall optical and optoelectronic properties^30,43,47,49–51^. We have demonstrated the scalable synthesis route to obtain highly crystalline RP perovskites, as well as structural, optical and optoelectronic properties of thin films of RP perovskites (CH)_2_(MA)_n−1_Pb_n_I_3n+1._. We report a highly tunable energy band gaps which vary from 2.51 eV to 1.94 eV with n = 1 to n = 4 respectively, giving RP perovskite crystals of yellow, red, brown, and black colors respectively (Fig. 1a). The occurrence of multiple exciton peaks in the absorbance and photoluminescence spectrums confirmed the formation of mixed phase. Photodetectors are fabricated in vertical configuration (FTO/RPP/Al) with CH based RP perovskites (n = 1–4) as absorbing layer. The transient photocurrent measurements have shown fast and stable photoresponse. An increasing trend is observed in the photocurrent density from 17 nA/cm^2^, for n = 1, to 70 nA/cm^2^ for n = 4, under no bias conditions. Current–voltage (I-V) characteristics performed under dark and illumination condition as well as transient photocurrent measurements under different bias for n = 4, (CH)_2_MA_3_Pb_4_I_13_ perovskite photodetector, revealed the semiconducting behaviour of the material. The ease of fabrication, structural flexibility, as well as interesting optical and optoelectronic properties highlight the application of these RP perovskites for various optoelectronic devices such as photodetectors, solar cells, LED etc.

## Results and discussion

The RP perovskites *(CH)*_*2*_*(MA)*_*n−1*_*Pb*_*n*_*I*_*3n*+*1*_ for *n* = 1, 2, 3 and 4 samples were prepared by using the reported synthesis route^14,23,52,53^. The detailed synthesis process is mentioned in the experimental section. Figure 1a show the schematic illustration of the crystal structure of RP perovskites *(CH)*_*2*_*(MA)*_*n−1*_*Pb*_*n*_*I*_*3n*+*1*_ for *n* = 1, 2, 3 and 4, where the number of inorganic lead iodide layers are shown to increase with the value of integer n, as discussed in the above section. Digital camera images of as-synthesised dry powder and the corresponding thin films of layered perovskites *(CH)*_*2*_*(MA)*_*n−1*_*Pb*_*n*_*I*_*3n*+*1*_ for *n* = 1, 2, 3 and 4 are shown in Fig. 1a. The occurrence of different colour thin films out of different *n* value perovskite solutions shows that these fabricated RP perovskite materials have different number of inorganic PbI_6_ monolayers in between the CH organic spacer monolayers. The change in *n* value also directly affects ratio of organic spacers (CH) and MA, which sits at the interstitial sites between the PbI_6_ octahedrons, which ultimately changes their energy band gap. Therefore, for this interesting system with CH as organic spacer, it becomes very attractive to examine how this cyclic organic moiety can affect their crystallographic and photo physical properties.

Thin film X-Ray diffraction (XRD) measurements, shown in Fig. 1b, represents highly crystalline nature of RP perovskite *(CH)*_*2*_*(MA)*_*n−1*_*Pb*_*n*_*I*_*3n*+*1*_* (n* = *1–4)* spin coated thin films. In Fig. 1b, c, for n = 1 (CH)_2_PbI_4_, the XRD pattern has shown a narrow and intense characteristic diffraction peak at 5.14° angle corresponding to the *(002)* planes, resulting in interlayer d-spacing of 17.2 Å. The occurrence of characteristic *(00l)* diffraction peaks signifies that *(00l)* planes are parallel to each other and are stacked along c-axis, thus forming a layered perovskite of orthorhombic crystal structure^23,54,55^. On comparison with the XRD pattern of (BA)_2_PbI_4_ thin film^49,56^ (see Supplementary Information Figure S1), the sharp characteristic diffraction peak *(002)* occurred at higher diffraction angle (6.40°), resulted in a smaller interlayer d-spacing of 13.8 Å, which is attributed to the small size of BA (linear carbon atoms chain) organic moiety, compare to CH (carbon atoms cyclic structure). For n = 2 RP perovskite, with the introduction of methylamine (MA), stoichiometric formula converts to (CH)_2_MAPb_2_I_7_ and intense diffraction peak corresponding to *(0k0)* planes were observed, showing the presence of 3D perovskite phase^57–59^. However, the occurrence of a low intense peak corresponding to (*002*) plane at diffraction angle 2θ ≈ 5.10^o^ (shown by dot) for n = 2, indicate that a pure 2D phase perovskite material is also present. For n = 3 RP perovskite (CH)_2_MA_2_Pb_3_I_10_, the XRD patterns thin film exhibit distinct peaks at 14.18° and 28.42° which can be ascribed to *(111)* and *(222)* crystallographic planes respectively which are present in the pure 3D perovskites of type CH_3_NH_3_PbI_3_^31^. Moreover, the occurrence of diffraction peaks of n = 2 in XRD pattern of n = 3 show the presence of mixed phases. The characteristic peak of the low-dimensional perovskite n = 1 eventually disappeared as the n-value increased from 2 to 4 as structure became more quasi-2D. For n = 4 RP perovskite (CH)_2_MA_3_Pb_4_I_13_, the XRD pattern shown intense diffraction peaks of 3D perovskite along with the pure phase of n = 4^22,60,61^. These thin film XRD studies indicate the formation of mixed phase highly crystalline RP perovskite films by the solution processing route. From the interlayer spacing calculations, using Bragg’s law, it was observed that with increase in the *n*-value (number of PbI_6_ inorganic layers) the resultant *d-spacing* also increases from 1.73 nm (for n = 1) to 2.42 nm (n = 4) (see Supplementary Information Figure S2 and Table S1).

**Figure 2:**
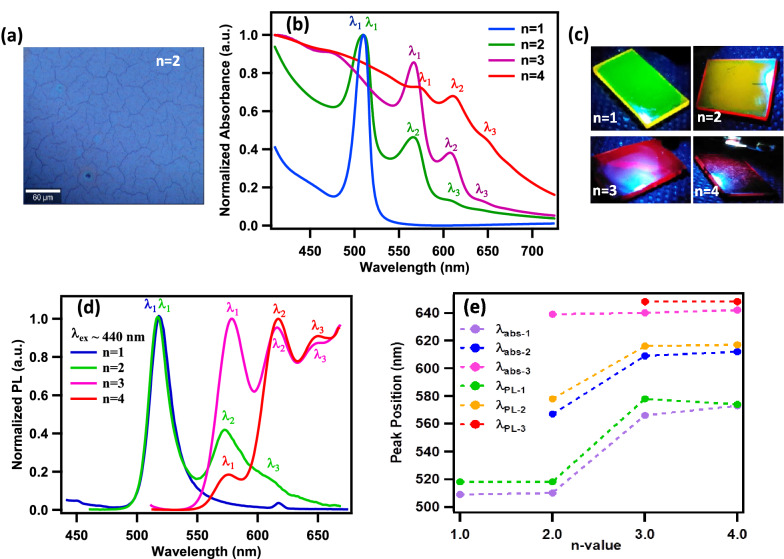
(**a**) Bright field optical microscope image of n = 2 hot casted thin film (scale bar 60 μm), (**b**) Room-temperature optical absorption spectrum of (CH)_2_(MA)_n−1_Pb_n_I_3n+1_ (n = 1, 2, 3 and 4) thin films, (**c**) Photoluminescence (PL) camera images of thin films of (CH)_2_(MA)_n−1_Pb_n_I_3n+1_ (n = 1, 2, 3 and 4) recorded by UV light source (300 nm LED) excitation and without using any long pass filter, (**d**) PL spectrum of (CH)_2_(MA)_n−1_Pb_n_I_3n+1_ (n = 1, 2, 3, and 4) thin films (λ_ex_ = 440 nm), (**e**) Variation of absorption and emission exciton peak wavelengths, extracted from (**b**) and (**d**), with *n*-value.

The bright-field optical microscope imaging has shown large sized (~ 30–40 μm) and well-connected perovskite grains in the hot-casted spin coated thin film of n = 2 RP perovskite sample (Fig. 2a). It must be noted that the hot-casting route is known to form large (~ above 10 micro-meters) size crystalline grains as well as pin-hole free films which makes it an ideal approach for device fabrication^62^.

The photo-physical properties of new *(CH)*_*2*_*(MA)*_*n−1*_*Pb*_*n*_*I*_*3n*+*1*_* n* = 1–4 thin films are studied by performing several optical characterizations and measurements. The UV–visible spectroscopy is used to record the optical absorption spectra of *(CH)*_*2*_*(MA)*_*n−1*_*Pb*_*n*_*I*_*3n*+*1*_* n* = 1–4 thin films (Fig. 2b). As expected the absorption spectra of (CH)_2_PbI_4_ (n = 1) demonstrated strong and sharp exciton absorption peak at 508 nm (green) with FWHM of 19 nm, which is attributed to the quantum and dielectric effects. Pure 2D metal halide perovskites (n = 1) resembles the multiple quantum wells (MQWs) type of structures where the inorganic layer of PbI_6_ octahedrons (thickness ~ 6.45 Å) form quantum well which are placed exactly between two bulky organic layers (thickness ~ 11.96 Å) forming quantum barriers, thus giving rise to strong quantum confinement effect that enable the formation of high binding energy excitons at room-temperature^63^. However, for higher member of the series (n > 1), the number of inorganic metal halide layer increases with the n-value and a small cation (MA) is also present in between interconnected PbI_6_ octahedron of the inorganic layers, giving rise to an inorganic layer of larger thickness but relatively smaller dielectric constant. Hence due to reduced dielectric constant and increase width of the quantum well region the overall dielectric and quantum confinement effect is weak which results in relatively smaller exciton binding energies. It must be noted that on comparison with the long carbon chain based organic (BA), the cyclic group organic (CH) has shown strong, sharp and intense exciton absorbance peak (Supplementary Information Figure S3). In the absorbance spectrum of *(CH)*_*2*_*(MA)*_*n−1*_*Pb*_*n*_*I*_*3n*+*1*_ n = 2–4 thin films a uniform red-shift in exciton peaks is observed with increase in n value. Moreover, in the absorption spectrums of n = 2–4 thin films, the presence of mixed RP phases is also observed. For instance for n = 2 (CH)_2_(MA)Pb_2_I_7_ film, an exciton absorption peak is observed at 567 nm (λ_2_) along with the characteristic absorbance peak corresponding to n = 1 at 508 nm (λ_1_) thus showing the presence of lower n value phase in the RP perovskites^64^. Similarly for n = 3 (CH)_2_MA_2_Pb_3_I_10_ film, exciton absorption peaks were observed at 568 nm (λ_1_), 608 nm (λ_2_) and 644 nm (λ_3_), while for n = 4 (CH)_2_MA_3_Pb_4_I_13_ film exciton absorption peak were observed at 572 nm (λ_1_), 610 nm (λ_2_) and 646 nm (λ_3_). For n = 3 and 4, the multiple exciton peak positions are very identical, though the peak intensities and linewidths are different. 568 nm (λ_1_) is intense peak in case of n = 3, however for n = 4, 610 nm (λ_2_) is an intense peak. The effective bandgap energy (E_g_) of *(CH)*_*2*_*(MA)*_*n−1*_*Pb*_*n*_*I*_*3n*+*1*_ for n = 1–4 thin films is obtained from the Tauc’s plot (Supplementary Information Figure S4). For n = 1 to n = 4, the E_g_ is found to decrease from 2.51 eV to 1.92 eV respectively. Further, the exciton binding energy (E_B.E._) is estimated using the relationship E_B.E._ ≈ (E_g_ – E_abs_) where E_abs_ is exciton absorption peak energy ^65–67^. The calculated exciton binding energies of *(CH)*_*2*_*(MA)*_*n−1*_*Pb*_*n*_*I*_*3n*+*1*_ thin films is found to be decreasing from 70 meV for n = 1 to 30 meV for n = 4, which is attributed to weak dielectric and quantum confinement effects as discussed above (See Table 1). It is observed that the exciton binding energies for higher member of series has become comparable with the 3D perovskite while still maintaining the quasi-2D structure^68^.

**Table 1:** Table showing the exciton absorbance and emission peak wavelengths for n = 1–4 thin films and the corresponding energy bandgap (**Eg**), Stokes’ shift parameter (**∆E)** and Exciton Binding Energy (**E**_**B.E.**_).

(CH)_2_(MA)_n−1_Pb_n_I_3n+1_	Abs peak λ_abs_ (nm)	E_abs_ (eV)	PL peak λ_PL_ (nm)	E_PL_ (eV)	Stokes shift (nm)	Stokes shift ∆E = E_abs_− E_PL_ (meV)	E_g_ (eV)	Exciton binding energy E_B.E._ = E_g−_E_abs_ (meV)
n = 1, (CH)_2_PbI_4_	508 (λ_1_)	2.44	518 (λ_1_)	2.39	10	50	2.51	70
n = 2, (CH)_2_(MA)Pb_2_I_7_	566 (λ_2_)	2.19	573 (λ_2_)	2.16	7	30	2.24	50
n = 3, (CH)_2_(MA)_2_Pb_3_I_10_	608 (λ_2_)	2.03	616 (λ_2_)	2.01	8	20	2.07	40
n = 4, (CH)_2_(MA)_3_Pb_4_I_13_	646 (λ_3_)	1.92	654 (λ_3_)	1.89	8	30	1.94	20

Photoluminescence (PL) camera images are obtained by illuminating the *(CH)*_*2*_*(MA)*_*n−1*_*Pb*_*n*_*I*_*3n*+*1*_ thin films using UV light source (LED, λex ~ 300 nm) under dark conditions and recording the images using CCD camera without using any long pass filter to block the UV light. The thin films have shown strong and tunable emission ranging from bright green for n = 1 to cherry red for n = 4 (Fig. 2c). To get further insights about the optical properties of thin films, the PL spectrums are obtained at room temperature using the Xe-lamp based excitation source (λ_ex_ = 440 nm) in PL spectrophotometer in reflection mode. As shown in Fig. 2d, a very intense and narrow exciton PL peak is observed for n = 1, with peak emission wavelength at 518 nm (λ_1_). Similar to the absorption spectra, multiple exciton emission peaks are observed for n = 2–4 value RP perovskite thin films covering wide range of emission wavelengths from 518 nm (λ_1_), 573 nm (λ_2_), 612 nm (λ_3_) for n = 2; 578 nm (λ_1_), 616 nm (λ_2_), 651 nm (λ_3_) for n = 3 to 573 nm (λ_1_), 617 nm (λ_2_), 654 nm (λ_3_) for n = 4. The variation of exciton peak wavelength with n-value for all exciton peaks observed in the absorbance and PL spectra show uniform and wide range exciton band tunability for *(CH)*_*2*_*(MA)*_*n−1*_*Pb*_*n*_*I*_*3n*+*1*_ thin films (Fig. 2e). The presence of intense emission at multiple wavelengths is also evident from the PL camera images of n = 2 and 3, where the top illumination of films by UV light has shown the emission corresponding to the longer wavelengths at the edges of the glass slide as waveguide effect.

The Stokes’ shift parameter (ΔE) is calculated using relationship, ΔE ≈ E_abs_ − E_PL_, where E_abs_ and E_PL_ are absorption and emission energy at exciton peaks (shown in Table 1). For *(CH)*_*2*_*MA*_*n−1*_*Pb*_*n*_*I*_*3n*+*1*_ thin films the characteristic absorbance and PL peaks corresponding to n = 1–4 are chosen (see Table 1 and Fig. 2e). Small values of Stokes’ shift ranging between 50 and 30 meV for n = 1–4 thin films shows the formation of highly defect free RP phase perovskite structures, which results in reduced non-radiative recombination losses, making these solution processed RP perovskite semiconductors a possible candidate for optoelectronic devices such as light-emitting diodes, lasers etc.

**Figure 3:**
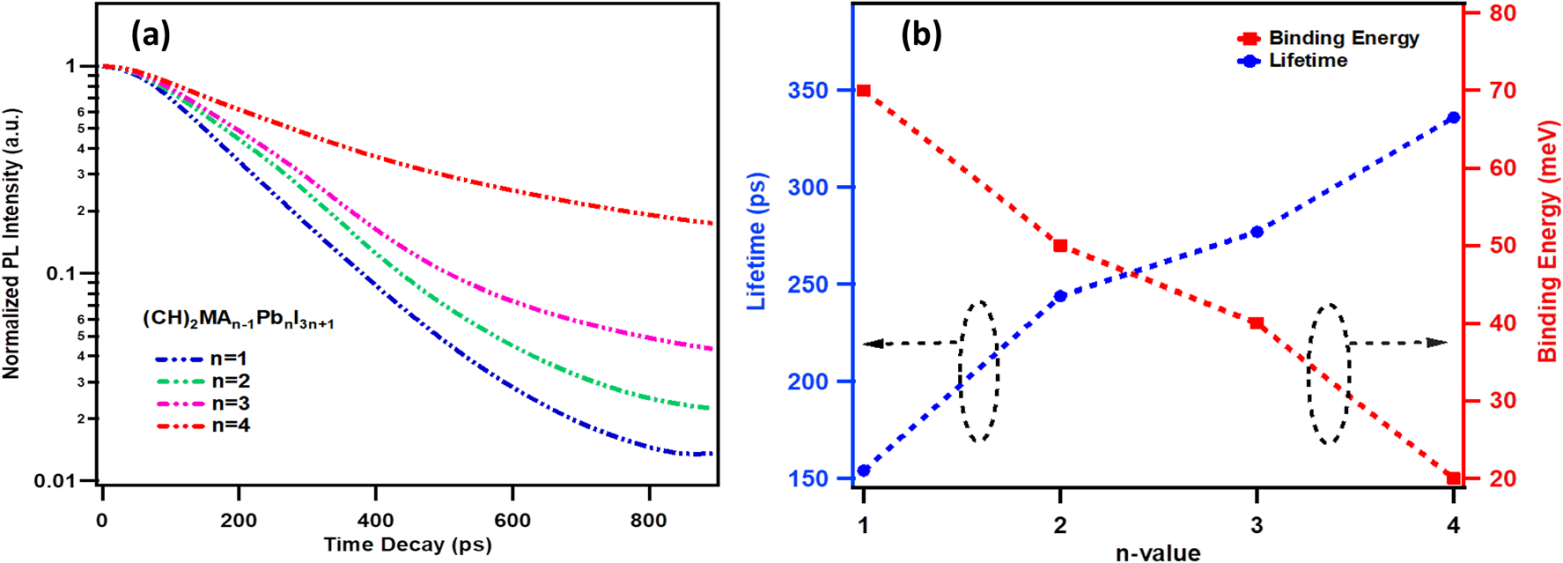
(**a**) The time resolved PL decay profiles of (CH)_2_(MA)_n−1_Pb_n_I_3n+1_ (n = 1, 2, 3 and 4) for perovskite thin films obtained by excitation of 401 nm pulsed diode laser. (**b**) Variation of corresponding charge-carrier recombination lifetime (left axis) and exciton binding energy (right axis) with n-value.

The charge carrier recombination lifetimes are measured for n = 1–4 thin films, spin coated on glass substrates, using the time correlated single photon counting (TCSPC) method. The thin film samples are mounted inside the integrating sphere and illuminated with pulsed diode laser of 401 nm excitation wavelength in the reflection mode to collect the emission signal. The emission signal is feed to the monochromatic coupled photo-multiplier tube (PMT) based picosecond photon detection (PPD) module. The time-resolved PL decay scans are performed at the characteristic emission peak wavelength for each *(CH)*_*2*_*(MA)*_*n−1*_*Pb*_*n*_*I*_*3n*+*1*_ for (n = 1, 2, 3, 4) perovskite thin film i.e., at λ_1_ for n = 1, at λ_2_ for n = 2, at λ_2_ for n = 3 and λ_3_ for n = 4. Figure 3a shows the time resolved PL decay curves for the n = 1–4 thin films collected at room-temperature. Double exponential curve fitting is used to estimate the charge carrier recombination lifetimes which has shown regular increase with n-value i.e., from τ_avg_ = 154 ps (n = 1), 244 ps (n = 2), 277 ps (n = 3) to 336 ps (n = 4) (Supplementary Information Table S2). Figure 3b shows variation of charge recombination lifetimes and corresponding exciton binding energies with the n-value. Tightly bound excitons (70 meV, n = 1) have shown lower value of lifetime (154 ps, n = 1) compare to loosely bound excitons (20 meV, n = 4) which has shown relatively large value of lifetime (336 ps, n = 4). The increase in the lifetimes and reduced exciton binding energy for higher n-value RP perovskites confirm that the quasi-2D perovskite phase is present in the RP perovskites and they possess 3D-perovskite like optoelectronic properties for higher members of the series, whereas for lower n-value members the optoelectronic properties are similar to the pure 2D perovskites.

**Figure 4:**
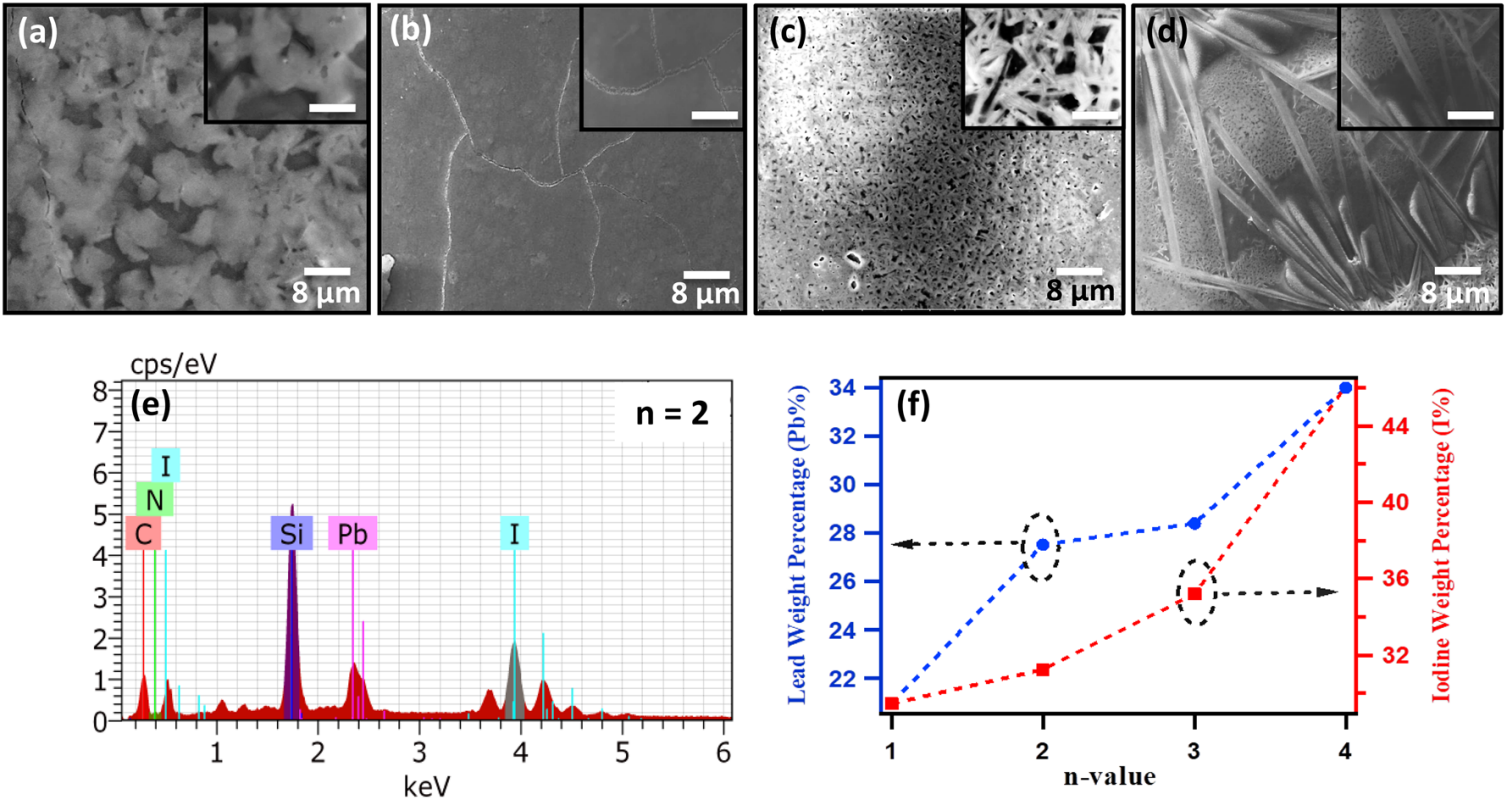
Top-view scanning electron microscope (SEM) images taken at 10 kX magnification for (**a**) n = 1, (**b**) n = 2, (**c**) n = 3 and (**d**) n = 4 thin films. Scale bar for all inset figures (**a**–**d**) is 1 μm. (**e**) Corresponding energy dispersive analysis (EDAX) spectra for n = 2 thin film. (**f**) Shows the variation in the elemental composition, obtained from EDAX, of iodine and lead for n = 1–4 RP perovskite thin films.

Scanning electron microscopy (FE-SEM) is performed on RP perovskites *(CH)*_*2*_*(MA)*_*n−1*_*Pb*_*n*_*I*_*3n*+*1*_ n = 1–4 thin films to study the surface morphology. All thin films are spin coated on the glass substrate from the DMF solution using hot-casting route. As shown in Fig. 4a-d, the top view SEM images shows grains of different morphology and size for n = 1–4 perovskite spin coated thin films. For n = 1, several surface pores are observed which may degrade the device performance for subsequent applications (Fig. 4a). For n = 2, highly smooth and large grains of size ~ 30–40 μm are observed, with very tightly packed grains which resulted in reduced area of the grain boundaries (Fig. 4b). Such arrangement of crystalline RP perovskite grains can be useful to enhance the device performance. It must be noted that for the hot-casting deposition route, the growth of large grains depends upon the uniformity of substrate temperature^62^. As the temperature of substrate is higher at the middle and lower at the edges, therefore large grains are observed in the middle portion of film, whereas partial as well as no grains were observed at the edges of the substrate (see Supplementary Information Figure S5a, b). For n = 3, SEM images shown highly porous mesh-like nanostructures of size ~ 50 nm, with good crystallinity and crystal orientation as confirmed by the XRD studies (Fig. 4c). Such mesh like architectures offers large surface area which improves the light absorbance. In 2018, Yongsheng and co-workers demonstrated highly oriented thin films of n = 3 (ThMA)_2_(MA)_2_Pb_3_I_10_ (ThMA = 2-thiophenemethylammonium) with mesh-like morphology which resulted in impressive improvement in the efficiency of perovskite photovoltaic devices from 1.74% to over 15%^69^. For n = 4, thin films displayed long nanorods like nanostructures of diameter ~ 80 nm and lengths ~ 50–60 μm, along with this the presence of porous mesh-like structures corresponding to n = 3 is also observed (Fig. 4d)^70^. The significantly distinct surface morphologies for all RP perovskites (n = 1–4) films is attributed to the presence of strong crystallographic orientations of *(002)* for n = 1, *(020)* for n = 2, *(020)* along with a low intensity *(111)* peak for n = 3, and *(111)* along with a low intensity *(020)* peak for n = 4, as shown by the X-ray diffraction studies (Fig. 1b). *(00l)* and *(0k0)* crystallographic orientations shows the parallel and perpendicular stacking of planes on the substrate^23,31,71^. The presence of dominant crystallographic orientation controls the overall surface morphology of the film, however the presence of other crystallographic orientations disturbs the film growth and limit the grain size.

Further, the elemental composition and their spatial distribution in the *(CH)*_*2*_*MA*_*n−1*_*Pb*_*n*_*I*_*3n*+*1*_ n = 1–4 thin films is verified by the energy dispersive X-ray (EDAX) analysis (see Supplementary Information Figure S6a for n = 2). Figure 4e shows the corresponding EDAX spectra, which confirms the presence of iodine and lead elements along with other elements (such as C,N) on the surface of the film, moreover the distribution of these elements is found to be very uniform across the scanning area (see Supplementary Information Figure S6b, c). As per the stoichiometric compositions of iodine and lead in RP perovskites, the atomic weight percentages are also found to increase as the n-value increase from n = 1 to n = 4 (Fig. 4f). It was observed that for n = 4 doped sample the elemental weight percentage of iodine (~ 46%) and lead (~ 34%) is found to be quite higher with respect to weight percentage of n = 3 Iodine (~ 36%) and lead (~ 28%), which is attributed to the presence of mixed phases.

**Figure 5:**
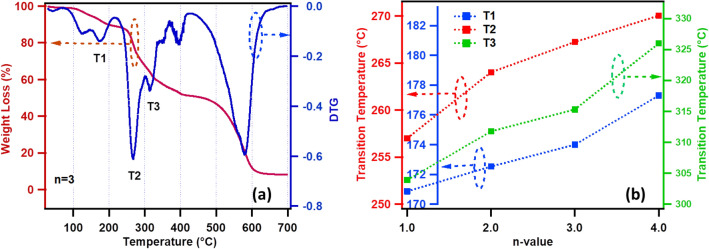
(**a**) Shows the thermogravimetric analysis (TGA) plot of (CH)_2_(MA)_n−1_Pb_n_I_3n+1_ for n = 3 powder sample with weight loss (%) on the left axis and first order differential thermogravimetric (DTG) on the right axis. (**b**) Variation of phase transition temperatures (T_1_, T_2_ and T_3_) with the n-value of RP perovskites.

To examine the thermal stability and phase transition temperatures of reported CH based RP perovskites *((CH)*_*2*_*(MA)*_*n−1*_*Pb*_*n*_*I*_*3n*+*1*_*)*, the TGA is performed on the dried powder samples. The samples were weighed in alumina crucibles, isothermally balanced for 15 min, and heated to 700 °C at a rate of 10 °C/min under a constant 20 ml min^−1^ N_2_ gas flow. Figure 5a shows the TGA plot obtained for n = 3 powder sample, the first phase transition is observed corresponding to the melting of organics (MA and CH) in the RP perovskite. As MA evaporates at lower temperature compare to CH therefore the first peak in the DTG curve represents the MA evaporation and second peak represents the CH evaporation, thus at 174 °C both organics decomposed completely (the higher transition temperature is termed as T_1_). Subsequently, structural phase transition (termed as T_2_) at 267 °C followed by the crumpling of the inorganic layers of Pb_3_I_10_ (termed as T_3_) at 315 °C are observed at relatively higher temperatures respectively^42^. Finally at much higher temperature (above 500 °C), the inorganic decomposition is observed. Similar trend of phase transitions and material decompositions are observed for all members of RP perovskite series *(CH)*_*2*_*(MA)*_*n−1*_*Pb*_*n*_*I*_*3n*+*1*_. However, with increase in n-value, the temperatures T_1_, T_2_, and T_3_ are found to increase linearly (see Fig. 5b, Supplementary Information Figure S7a–d and Table S3). We found that T_1_, T_2_, and T_3_ transition temperatures are increased by ~ 6.5 °C, 13.0 °C, and 22.0 °C for n = 4 compare to n = 1, as shown in Fig. 5b. It must be noted that large increase in T_3_ for n = 1–4 is due to more amount of inorganic material in the stoichiometry. Such increase in the phase transition temperatures shows that CH based RP perovskites show promising thermal and structural stabilities compare to previously reported BA (Butyl amine) or iBA (iso-butyl amine) based RP perovskites^40,41^.

**Figure 6:**
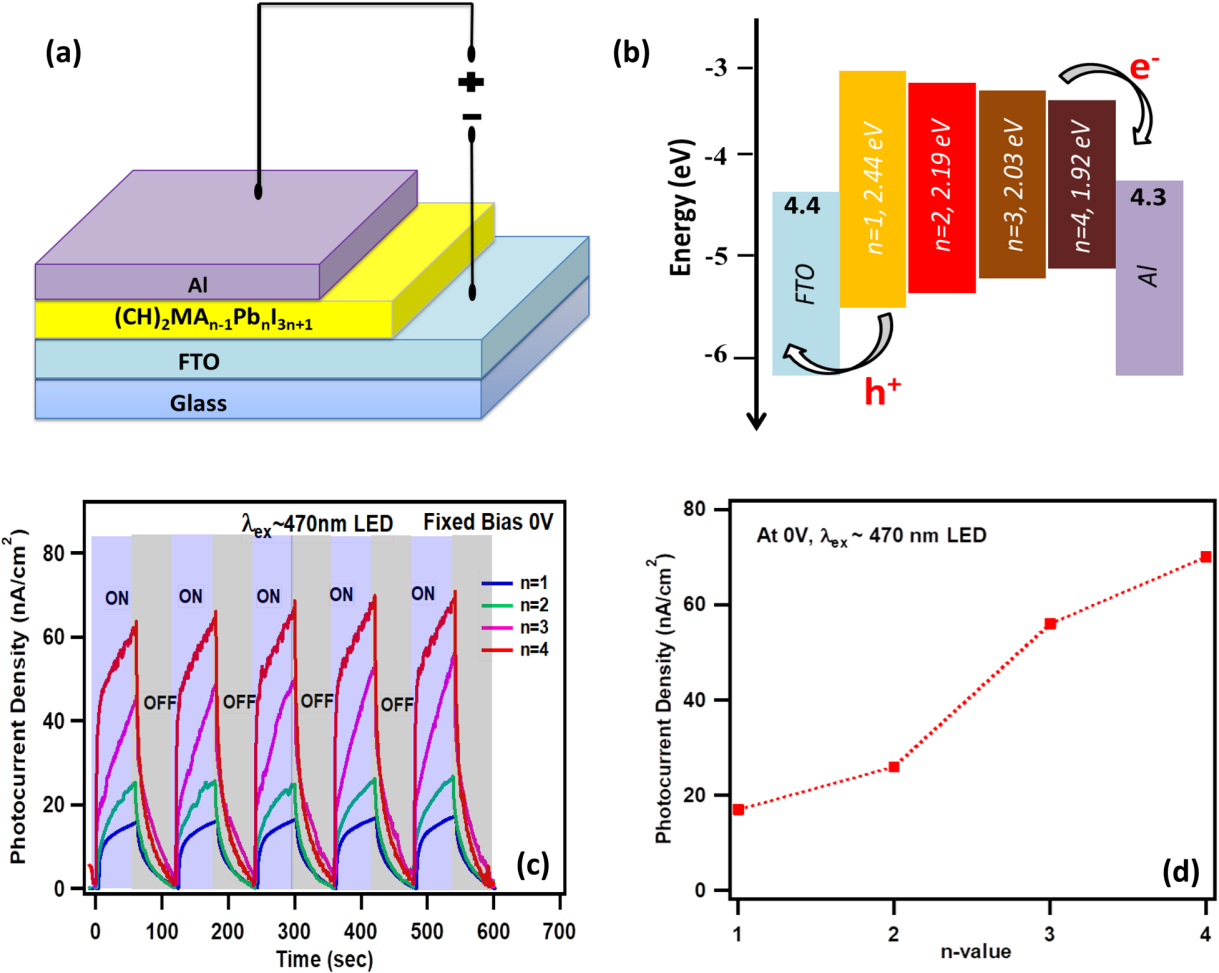
(**a**) Schematic illustration of fabricated (CH)_2_(MA)_n−1_Pb_n_I_3n+1_ perovskite photodetector. (**b**) Corresponding energy level diagram of photodetector shown in (**a**). (**c**) Transient photocurrent measurements (λ_ex_ = 470 nm LED) for n = 1–4 photodetector with under zero bias condition. (**d**) Variation of peak photocurrent density with n-value at 0 V.

Further to understand the effect of bandgap tunability on the optoelectronic properties of quasi-2D *(CH)*_*2*_*(MA)*_*n−1*_*Pb*_*n*_*I*_*3n*+*1*_ (n = 1–4) perovskites their photocurrent response is studied by fabricating vertical configuration (FTO/RPP(n = 1–4)/Al) photodetectors without using any electron and hole transport materials as shown in Fig. 6a (see experimental section for photodetector fabrication). Photocurrent measurements are performed by using a potentiostat and/or source meter unit and a low power blue LED light (λex = 470 nm, power density ~ 1.5 mW/cm^2^) source is used as an excitation source (see experimental section). The transient photocurrent are performed under dark conditions by turning ON and OFF the illumination every 60 s by using a manual shutter, thus producing a square wave-like pulsed excitation. The transient photocurrent response for n = 1–4 RP perovskite photodetectors recorded under zero bias has shown very fast rise in the photocurrent upon illumination in the beginning followed by a slow rise (Fig. 6b). For n = 1, rise time is 12 s for increase in photocurrent from 0.1 to 1.21 nA, such increase in the current immediately upon illumination indicate formation of large number of electrons-holes pairs in the RP perovskite semiconducting thin film which subsequently got dissociated and travelled to their respective current collector electrodes (as shown in Fig. 6c) and readily gets extracted. When the LED light was turned OFF, a similar fast decay in the current is observed in the beginning followed by a slow decay. For n = 1, decay time is 18 s for decay in current from 1.30 nA to 0.3 nA, such decay in current is due to the fast collection of charge carriers in the immediate vicinity of contacts, whereas slow decay in the current afterwards is due to shielding effect due to the accumulation of large number of charge carriers^72,73^. As we reported earlier, the 470 nm excitation induce the band to band transition in the RP perovskites, which in turn produces free charge carriers however in order to generate exciton photocurrents, the excitation energy must be chosen as per the energy of exciton band^10,41,74^. Also, it is important to note that with laser illumination of appropriate energy a large and ultrafast transient photocurrent response can be obtained due to the generation of substantially large number of free charge carriers compare to low power LED based illumination.

A linear increase in the photocurrent is observed as the n-value is increased from n = 1 (17 nA/cm^2^) to n = 4 (70 nA/cm^2^) under zero bias and similar illumination conditions (Fig. 6d). Such increase in photocurrent with n-value is due to the reduction in the energy band gaps (E_g_) with increase in n-value of the RP perovskites (see Fig. 2b and Table 1). The responsivity of photodetectors are calculated for blue light illumination (470 nm LED, 1.5 mW/cm^2^) at 0 V using formula ${R=(I}_{light}-{I}_{dark})/(Pin*A),$ where ${I}_{light}$ is the light current, ${I}_{dark}$ is the dark current, $Pin$ is the optical power intensity and $A$ is the active area. The responsivities are found to be 11.30 mA/W (for n = 1), 17.35 mA/W (for n = 2), 37.33 mA/W (for n = 3) and 46.65 mA/W (for n = 4). Encouragingly, n = 4 device shows the highest responsivity among all n-values owing to the lowest energy bandgap (~ 1.94 eV), relatively large charge carrier lifetime (336 ps) and lowest exciton binding energy (20 meV).

**Figure 7:**
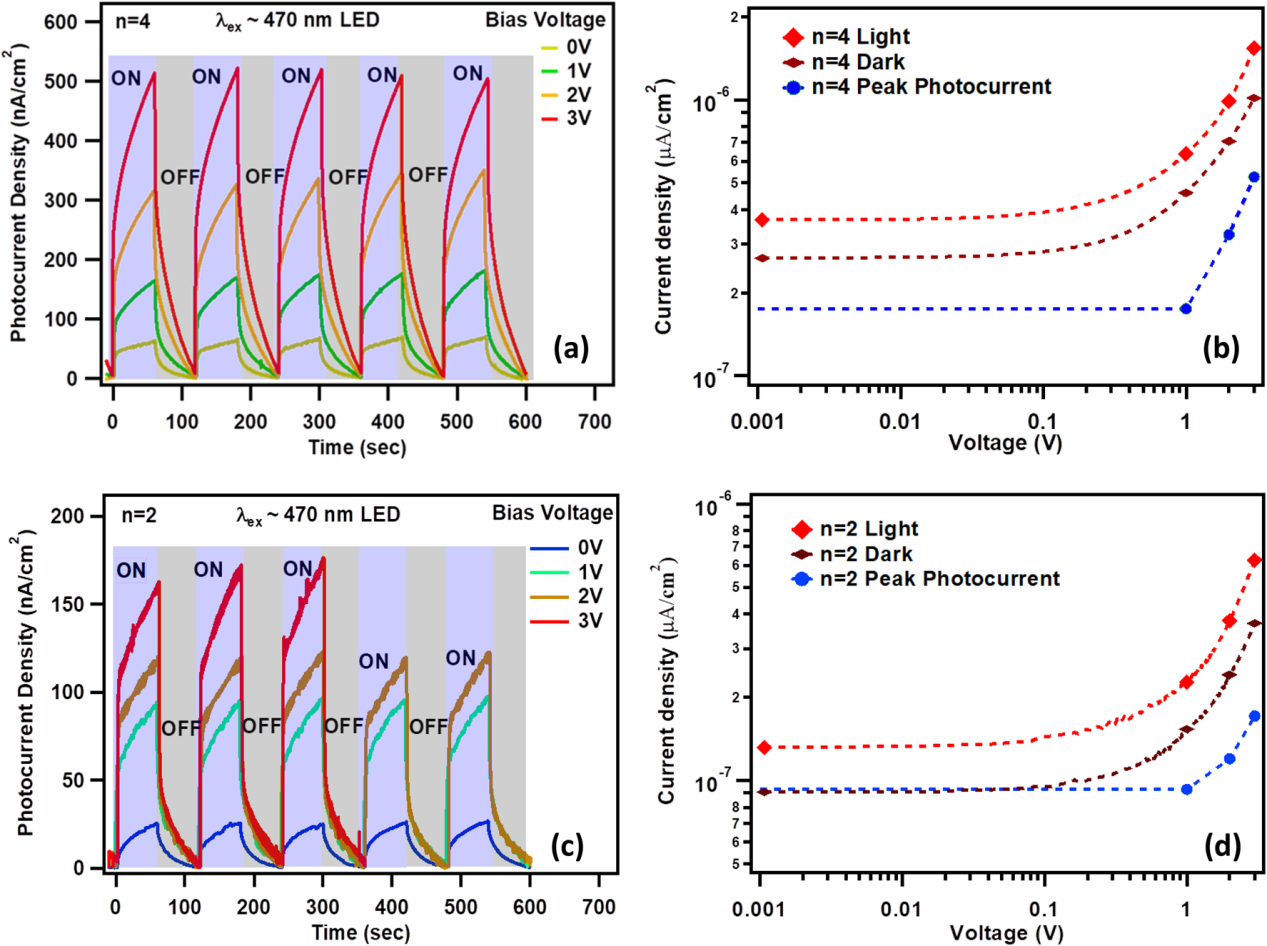
Transient photocurrent (λ_ex_ = 470 nm LED) for (**a**) n = 4 and (**c**) n = 2 RP perovskite photodetector measured under different voltage bias of 0.0 V to + 3.0 V. Current density–Voltage (J–V) curve measured from −3.0 to + 3.0 V for (**b**) n = 4 and (**d**) n = 2 RP perovskite photodetector measured under similar illumination condition. For comparison the peak photocurrent densities extracted from the transient photocurrent measurements (blue dotted curve) are added.

Due to low energy band gap and other above-mentioned properties we further investigated n = 4 RP perovskite photodetectors. Transient photocurrent measurements are performed for n = 4 photodetectors under applied bias of 0.0 V to + 3.0 V and similar illumination condition (LED, λ_ex_ ~ 470 nm). Peak photocurrent densities of 508 nA/cm^2^ at 3.0 V, 350 nA/cm^2^ at 2.0 V, 180 nA/cm^2^ at 1.0 V and 70 nA/cm^2^ at 0.0 V are obtained for n = 4 photodetector (Fig. 7a). These peak photocurrent densities when plotted against the applied voltage bias shows an exponential rise in the photocurrent with bias which revealed the semiconducting behavior of the reported CH based RP perovskites (n = 4) (Fig. 7b). The semiconducting behavior is further confirmed by performing I-V measurements from −3.0 V to + 3.0 V, which has shown typical diode-like characteristics (Fig. 7b). Similar transient photocurrent and IV measurements are also performed for n = 2 based photodetector and shown enhancement in peak photocurrent density from 26 nA/cm^2^ at 0.0 V to 171 nA/cm^2^ at 3.0 V. (Fig. 7c, d). It must be note that the power exponent factor *m* (power law), obtained from the I–V curves, for n = 4 (m ~ 0.80) and n = 2 (m~ 0.93) have indicated ohmic conduction in the low voltage region.

## Conclusion

Cyclic ring organic 2-(1-Cyclohexenyl)ethylamine (C_6_H_9_C_2_H_4_NH_2_^+^) is used an organic spacer to form Ruddlesden–Popper phase perovskites of type (CH)_2_(MA)_n-1_Pb_n_I_3n+1_ (n = 1–4) and are investigated for optoelectronic applications. Thin film optical absorbance revealed that their energy bandgap varies from 2.51 eV (green) to 1.92 eV (red) with change in the n-value, number of inorganic PbI_6_ layers, from n = 1 to n = 4 in the RP perovskite crystal. The cyclic organic spacer in the RP perovskite structure has improved the confinement effects which has enabled the formation of sharp and intense PL at room temperature for all thin films (n = 1–4). TCSPC measurements on thin film samples confirmed that the photo-generated charge carrier recombination lifetimes increased from 154 to 336 ps with change in n-value from n = 1 to n = 4 respectively. Further SEM imaging showed that the surface morphology of *(CH)*_*2*_*(MA)*_*n-1*_*Pb*_*n*_*I*_*3n*+*1*_ thin films is highly dependent upon the n-value. EDAX measurements confirm uniform distribution of elements (Pb and I) on the film surface with varied uniformly with change in the stoichiometry for n = 1–4. Inert atmosphere TGA studies on the powder samples highlighted very impressive structural and thermal stability of CH based RP perovskites. Transient photocurrent measurements performed on photodetectors with (CH)_2_(MA)_n−1_Pb_n_I_3n+1_ (n = 1–4) RP perovskite as light absorbing layer showed dependency of photocurrent on the number of inorganic sheets and applied bias. An enhancement in photocurrent density from 17 to 70 nA/cm^2^, under zero bias and 470 nm blue LED illumination, for n = 1 to 4 photodetector respectively even for Al-tape as metal contacts and without any charge transport layer is very promising. n = 4 perovskite showed highest photocurrent compare than lower member of series reaching 508 nA/cm^2^ at 3 V. Overall the cyclic ring type organic spacer cation CH based RP perovskites has shown improved optoelectronic properties such as tunable energy band gaps, high carrier mobilities and charge carrier lifetimes, as well as promising structural stability which makes them a potential candidate to be explored for the development of advanced optoelectronic devices such as photodetector, solar cells, LEDs, photobatteries etc.

## Experimental Section

### Required chemical

Lead oxide (PbO, 99%), Methylammonium chloride (CH_3_NH_3_Cl, 99%), 2-(1-Cyclohexenyl)ethylamine (C_6_H_9_C_2_H_4_NH_2_^+^, ≥ 98%), Hydriodic acid (HI, 57 wt % in H_2_O), hypophosphorous acid (H_3_PO_2_, 50 wt.% in H_2_O), N,N-dimethylformamide (DMF, anhydrous, 99.8%), were used as received from Sigma-Aldrich.

### Synthesis of ***(CH)***_***2***_***(MA)***_***n-1***_***Pb***_***n***_***I***_***3n***+***1***_*** (n*** = ***1–4)***

PbO (5 mmol) was dissolved in 57% w/w aqueous HI solution (6 ml, 38 mmol) with 50% aqueous H_3_PO_2_ (850 µl, 7.75 mmol) by heating to boiling under constant magnetic stirring for 10 min. After the hot PbI_2_ solution turned into bright yellow solution, MACl (2.5 mmol for *(CH)*_*2*_*(MA)PbI*_*7*_* (n* = *2)*, 3.33 mmol for *(CH)*_*2*_*(MA)*_*2*_*Pb*_*3*_*I*_*10*_* (n* = *3)* and 3.75 mmol for *(CH)*_*2*_*(MA)*_*3*_*Pb*_*4*_*I*_*13*_* (n* = *4)*) is added subsequently and the yellow solution showed the black precipitate formation, which were redissolved under stirring and reflux condition at heating to boiling. Note that for n = 1, MACl was not added to the hot PbI_2_ solution. In a separate beaker, 2-(1-Cyclohexenyl)ethylamine (5 mmol for *(CH)*_*2*_*PbI*_*4*_* (n* = *1)*, 3.5 mmol for *(CH)*_*2*_*(MA)Pb*_*2*_*I*_*7*_* (n* = *2)*, 1.67 mmol for *(CH)*_*2*_*(MA)*_*2*_*Pb*_*3*_*I*_*10*_* (n* = *3)* and 1.25 mmol for *(CH)*_*2*_*(MA)*_*3*_*Pb*_*4*_*I*_*13*_* (n* = *4)*) was neutralized with HI 57% w/w (3 mL, 22.8 mmol) at room temperature giving pale yellow precipitates, which were dissolved under stirring and heating. Next, the 2-C_6_H_9_C_2_H_4_NH_3_I solution is mixed with the hot PbI_2_ solution under constant stirring and heating and 7.5 mmol 57% w/w aqueous HI solution is added for proper mixing. The magnetic stirring and heating were stopped all four mixed solutions (for n = 1–4) were left overnight for the precipitation, during the cooling phase, different colored RP perovskite crystals started crystallize for different n-value. For *(CH)*_*2*_*PbI*_*4*_* (n* = *1)* orange, for *(CH)*_*2*_*(MA)Pb*_*2*_*I*_*7*_* (n* = *2)* red, for *(CH)*_*2*_*(MA)*_*2*_*Pb*_*3*_*I*_*10*_* (n* = *3)* deep red/purple and *(CH)*_*2*_*(MA)*_*3*_*Pb*_*4*_*I*_*13*_* (n* = *4)* black colour rectangular shaped crystals were obtained, which were extracted later by using conventional route.

### Thin film and photodetector fabrication

The glass substrates were washed and sonicated in acetone and IPA respectively 15 min each followed by O_2_ plasma etching (10 min). Hot-casting spin coating route is followed to deposit thin films in ambient air conditions^62^. Briefly, the glass substrate is heated to 130 ˚C and the DMF solution of perovskite is heated to 70 ˚C, and spin-coating is performed at 1000 rpm for 5 s by quickly mounting the glass substrate on the spin coater chuck, followed by a drying step of 3000 rpm for 40 s. The resultants thin films were annealed at 100 °C for 10 min on the hot plate. The photodetectors (n = 1–4) were fabricated on fluorine doped tin oxide (FTO) coated glass substrates (12 × 12 mm^2^, 2.3 mm, ~ 13 ohms/sq). FTO is patterned in a strip shape (12 × 3 mm^2^) by using chemical etching route. After etching, FTO substrates were washed by soap and further cleaned by ultra-sonication in deionized water, acetone and IPA for approximately 15 min each. Just before spin coating the FTO substrates were treated with oxygen plasma for 10 min. The thin films of RP perovskite are deposited using the DMF solution of perovskite (0.5 mol/L) at 500 rpm for 5 s followed by 3000 rpm for 30 s under ambient atmospheric conditions. The film is later annealed on hotplate at 100 °C for 10 min. For the bottom metal contact, one-sided aluminum tape strip (12 × 3 mm^2^) is placed on top of the perovskite film and pressed well to avoid any gap, keeping the device active area fixed to 9 mm^2^. Similar procedure is followed for all n-value perovskites. The ‘Characterizations’ sub-section is available in the Supplementary Information file.

## Supplementary Information

Supplementary Information.
