# Supplementary material for: Ruddlesden–Popper 2D perovskites of type (C6H9C2H4NH3)2(CH3NH3)n−1PbnI3n+1 (n = 1–4) for optoelectronic applications
Source: Sci Rep. 2022 Feb 9;12:2176. doi: 10.1038/s41598-022-06108-8 (PMC8828857; doi:10.1038/s41598-022-06108-8)
Supplement: Supplementary file 1 — Supplementary Information. [file 41598_2022_6108_MOESM1_ESM.pdf]

Supporting Information

Ruddlesden-Popper 2D perovskites of type $(C_6H_9C_2H_4NH_3)_2(CH_3NH_3)_{n-1}Pb_nI_{3n+1}$ (n=1-4) for Optoelectronic Applications

Mohammad Rahil[1,2], Rashid Malik Ansari[1], Chandra Prakash[3], S. S. Islam[2], Ambesh Dixit[3] and Shahab Ahmad[1,*]

[1] Advanced Energy Materials Group, Department of Physics, Indian Institute of Technology Jodhpur, 342037 Jodhpur, Rajasthan, India.

[2] Centre for Nanoscience and Nanotechnology, Jamia Millia Islamia (A Central University), New Delhi 110025, India.

[3] Department of Physics, Indian Institute of Technology Jodhpur, 342037 Jodhpur, Rajasthan, India.

Corresponding Author: shahab@iitj.ac.in

Characterizations

X - ray diffraction is performed using XRD Smart Lab Guidance, Rigaku with Cu Kα radiation (λ = 1.54056 A) in step size of 0.02˚ and Bragg's angle range of 2θ= 3°-60° at glancing angle mode. SEM and EDAX are performed using FEI Nova NanoSEM 450 . TGA measurements were performed using STA 6000, Perkin Elmer with equipment control from Pyris manager and 0.1 microgram sensitivity. FTIR is performed using Vertex 70V, Bruker. UV-Vis spectroscopy is performed using UV-Vis Carry Series, Agilent Technologies and room temperature PL spectra were collected by Fluorlog 3 Flourometer, Horiba, equipped with 450 W Xenon lamp. PL lifetimes were analyzed by Delta Flex fluorescence spectroscopy TCSPC set-up, Horiba. The photocurrent measurements were performed using Autolab Potentiostat (Model No. AUT86243) in two-electrode configuration and a blue LED (470nm, 1.5 mW/cm^2), is used as an excitation source for photocurrent measurements. A low-power UV LED (300 nm) light source is used for PL camera imaging. Kiethley source meter unit (SMU 2450) is used to perform IV (current-voltage) measurements in dark and light.

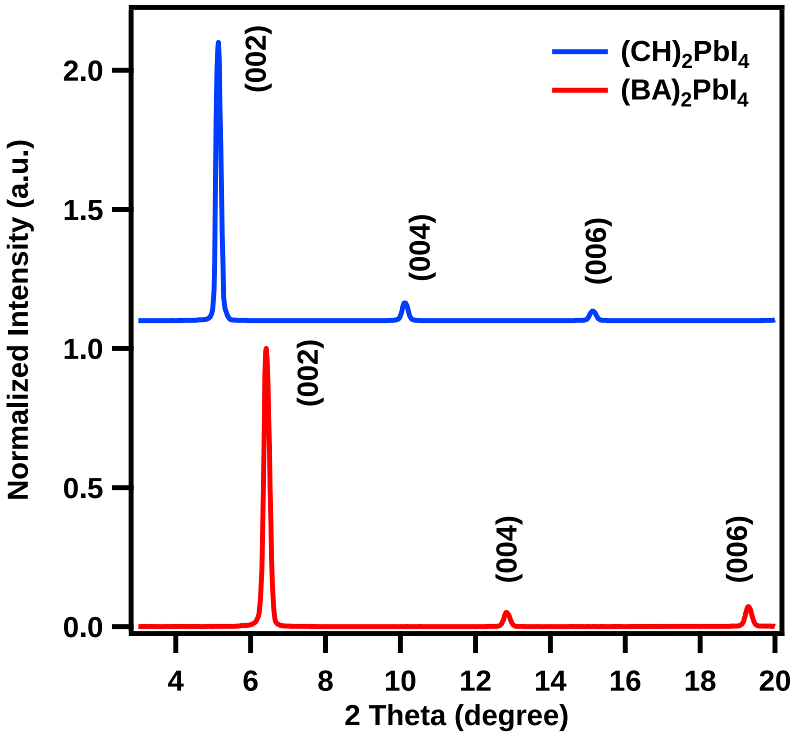

Figure S1: Comparison of characteristic X-ray diffraction peak (002) for pure 2D n=1 $(BA)_2PbI_4$, Butylammonium lead Iodide and n=1 $(CH)_2PbI_4$, Cyclohexnylethylammonium lead Iodide perovskite thin films.

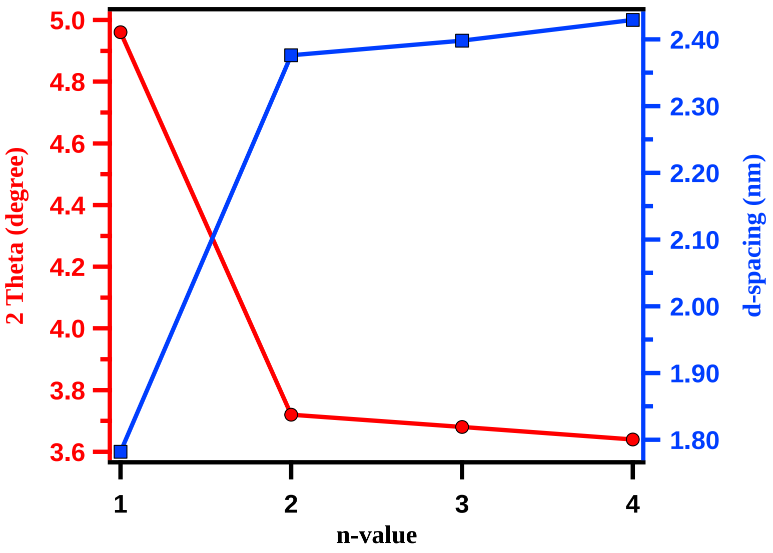

Figure S2: Variation in the diffraction angle 2θ (red line) and corresponding change in the interplanar *d*-spacing (blue line) with respect to the n-value in $(CH)_2(MA)_{n-1}Pb_nI_{3n+1}$ thin films

n-value	Plane	2 Theta (deg)	d spacing (nm)
n=1	*(0 0 2)*	5.14	1.72
n=2	*(0 2 0)*	3.72	2.376
n=3	*(0 2 0)*	3.68	2.398
n=4	*(0 2 0)*	3.64	2.429

Table S1. Shows the characteristic *hkl* plane and corresponding *d*-spacing for n=1-4 $(CH)_2(MA)_{n-1}Pb_nI_{3n+1}$ perovskite thin films.

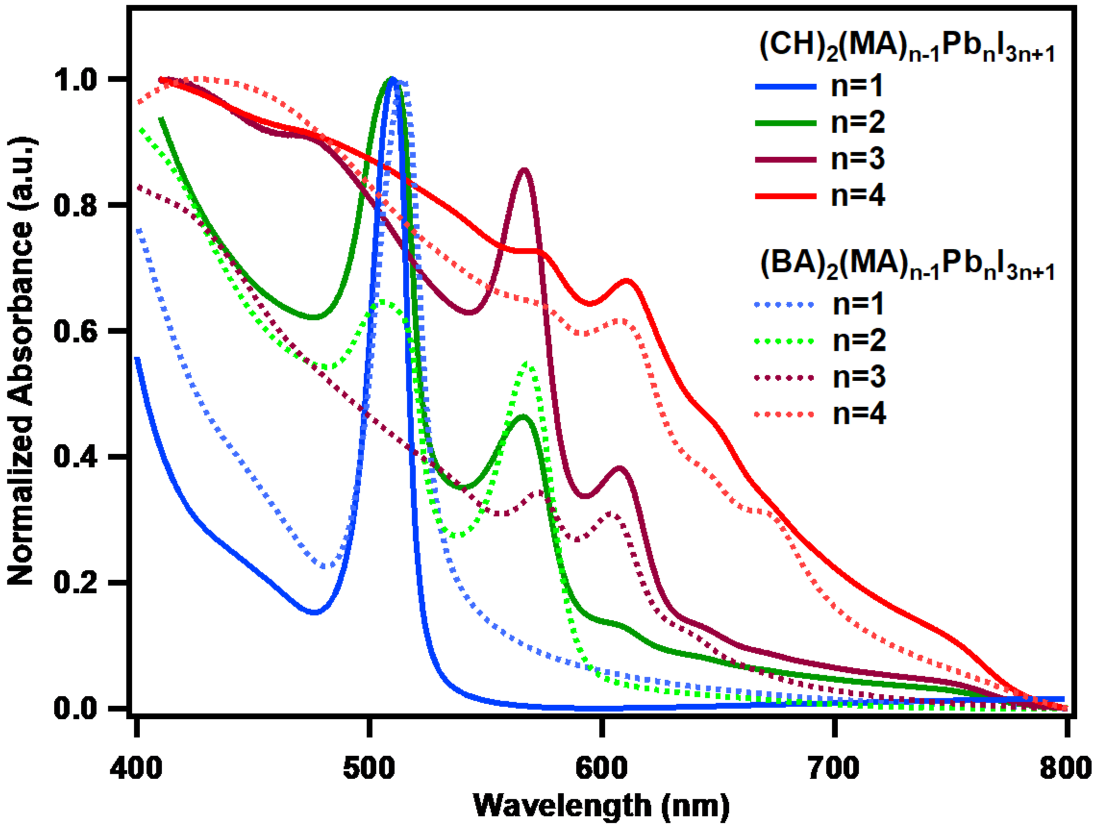

Figure S3. Comparison of optical absorbance for $(BA)_2(MA)_{n-1}Pb_nI_{3n+1}$ and $(CH)_2(MA)_{n-1}Pb_nI_{3n+1}$ n=1-4 thin films.

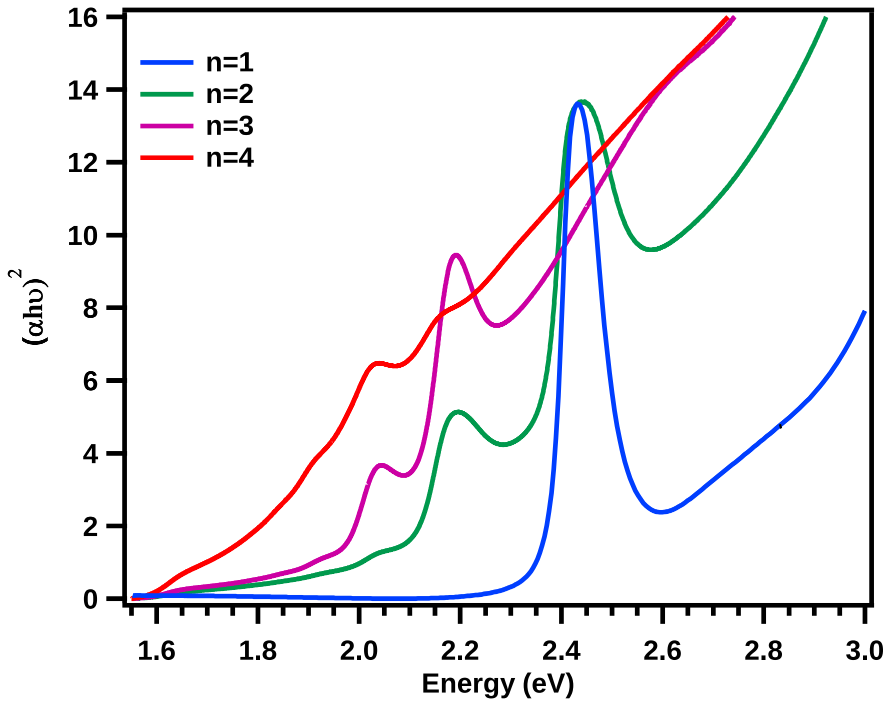

Figure S4: Tauc's plots, for band gap calculation, obtained from the thin film optical absorption spectrum of $(CH)_2(MA)_{n-1}Pb_nI_{3n+1}$ n=1-4.

value	A1	τ_1 (ps)	A2	τ_2 (ps)	τ_{avg} (ps)
n=1	0.52	150	0.58	160	154
n=2	0.66	237	0.51	253	244
n=3	0.59	193	0.63	324	277
n=4	0.77	293	0.11	134	336

Table S2. Time decay values τ_1 and τ_2, corresponding values of A1 and A2 factors respectively and the τ_{avg} values for $(CH)_2(MA)_{n-1}$ Pb_nI_{3n+1} n=1-4 thin films obtained from the double exponential curve fitting of TCSPC data.

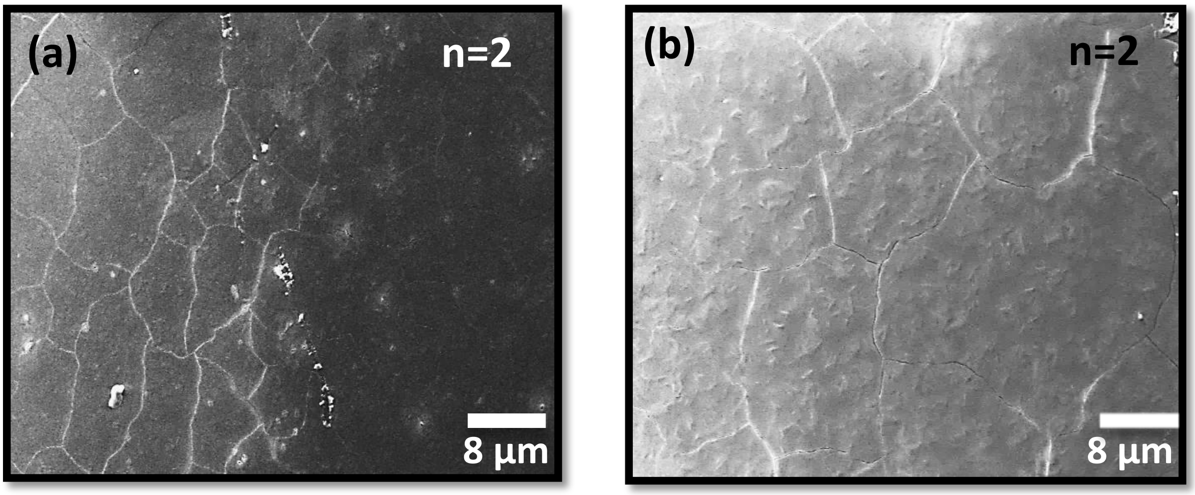

Figure S5: SEM image of n=2, $(CH)_2(MA)_{n-1}Pb_nI_{3n+1}$ hot-casted thin film. (a) SEM image taken at the edge of the film showing partially formed large grains of perovskite crystalline thin film during hot-casting due to temperature gradient. (b) SEM image taken at the centre of the film showing fully grown large size grains.

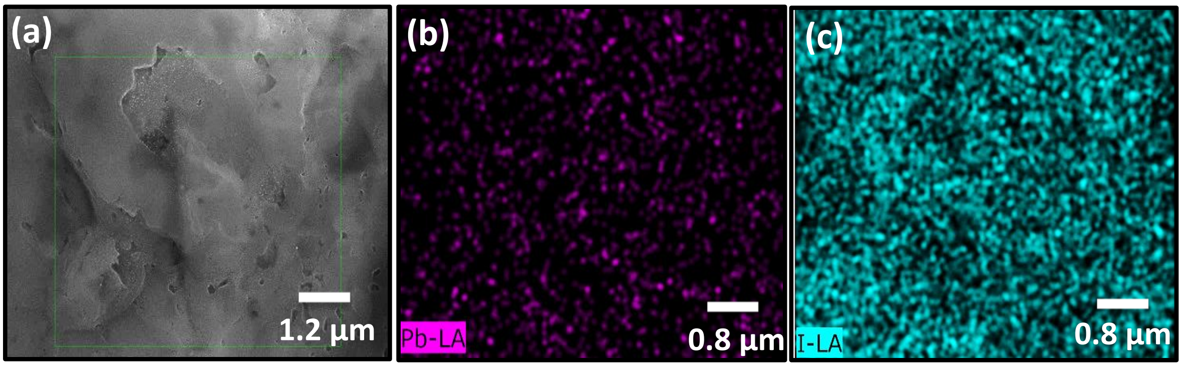

Figure S6: Thin films are deposited on glass substrate and SEM are recorded without any metal coating. The elemental mapping is performed over an area of 7 µm x 7 µm for all films. (a) SEM image of n=2 thin film showing scanned area, marked by green square, for elemental mapping. Corresponding elemental map for (b) Pb^{+2} and (c) I^- elements.

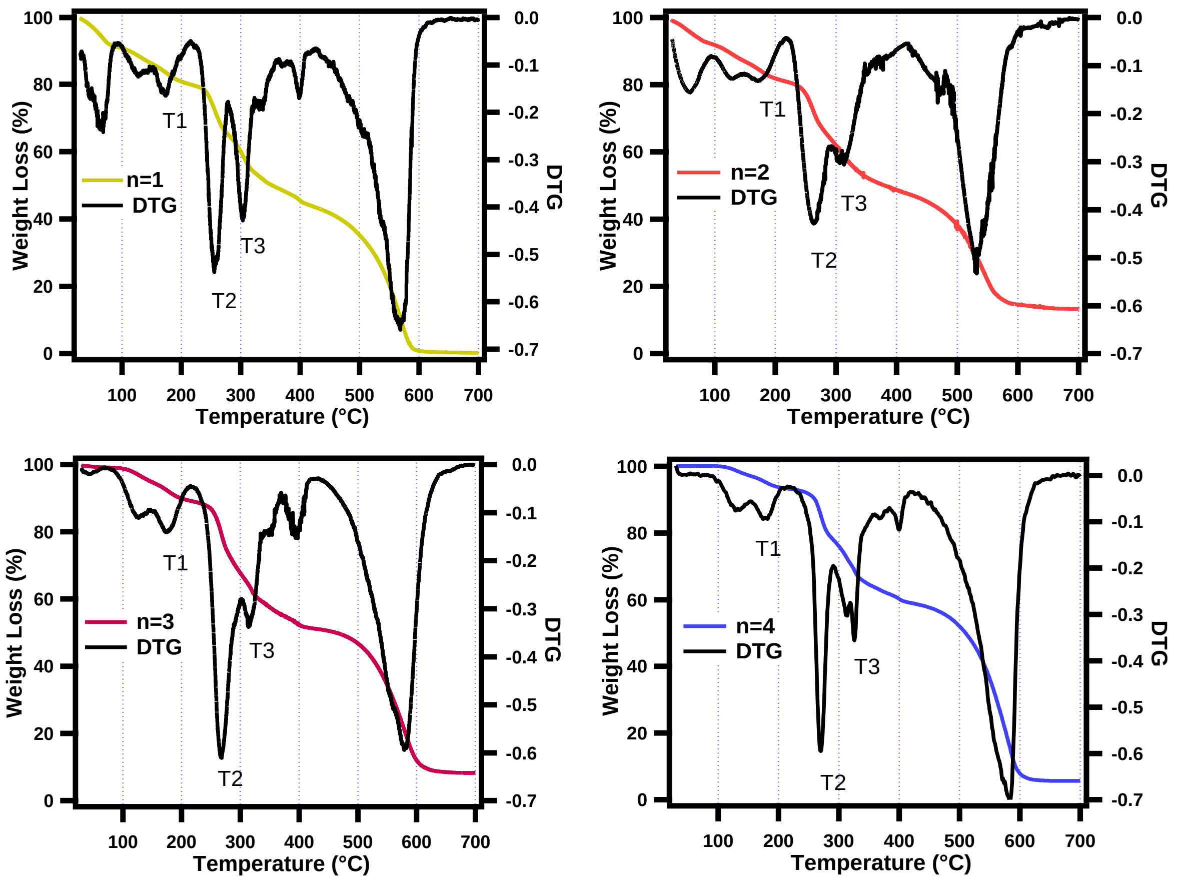

Figure S7: Thermogravemtric analysis (weight loss %) curve for *$(CH)_2(MA)_{n-1}Pb_nI_{3n+1}$* (n = 1, 2, 3, and 4) powder samples (left axis). Corresponding differential thermogravimetric (DTG) curve is plotted on the right axis of each plot.

n-value	T_1 (^{o}C)	T_2 (^{o}C)	T_3 (^{o}C)
**1**	**170.86**	**257**	**303.91**
**2**	**172.53**	**264.02**	**311.76**
**3**	**173.99**	**267.23**	**315.3**
**4**	**177.29**	**270.03**	**325.98**

Table S3: Transition temperature T_1,T_2 and T_3 extracted from DTG curves for *$(CH)_2(MA)_{n-1}Pb_nI_{3n+1}$* (n = 1, 2, 3, and 4) powder samples.

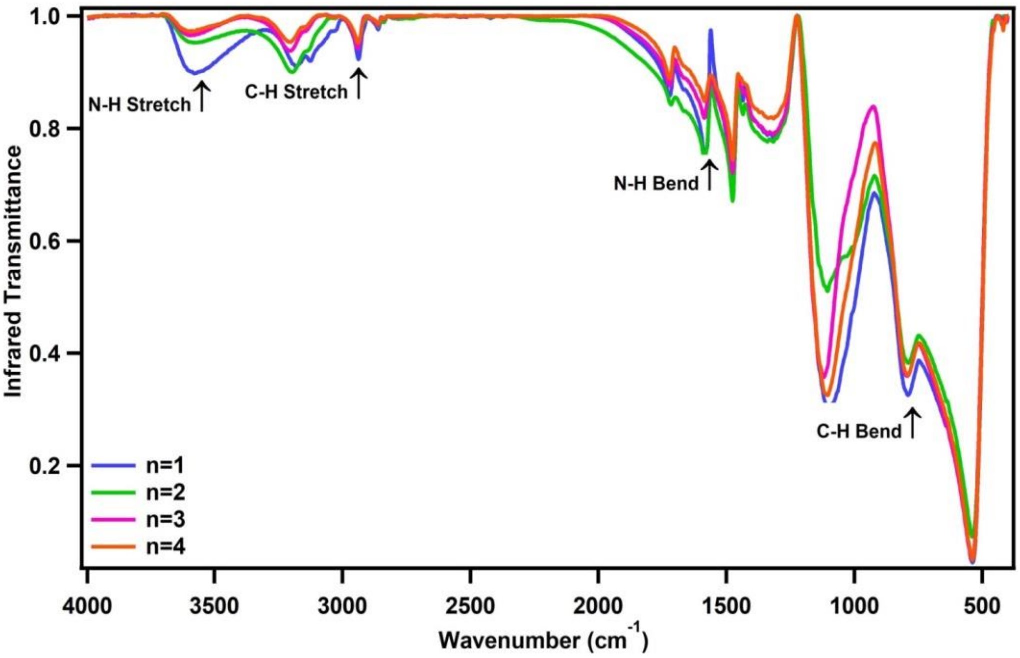

Figure S8. Fourier transform infrared (FTIR) spectrum of *$(CH)_2(MA)_{n-1}Pb_nI_{3n+1}$* (n = 1, 2, 3, and 4) thin films deposited on glass substrate.

References

1. Stoumpos, C. C. *et al.* Ruddlesden-Popper Hybrid Lead Iodide Perovskite 2D Homologous Semiconductors. *Chem. Mater.* **28**, 2852–2867 (2016).
2. Ahmad, S. *et al.* Strong Photocurrent from Two-Dimensional Excitons in Solution-Processed Stacked Perovskite Semiconductor Sheets. *ACS Appl. Mater. Interfaces* **7**, 25227–25236 (2015).
